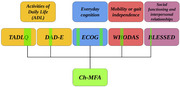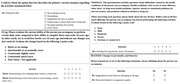# A machine learning approach for a multidimensional analysis of functional phenotype allows for a better measurement of dementia and its severity

**DOI:** 10.1002/alz70857_107014

**Published:** 2025-12-26

**Authors:** Cristian Muñoz, Nilton Custodio, Rosa Montesinos, Belen Custodio, Diego Bustamante‐Paytan, Carmen Dominguez, Álvaro Gallardo, Cecilia Gonzalez Campo, Patricia Lillo, Gonzalo Farías, Mauricio Cerda, Andrea Slachevsky

**Affiliations:** ^1^ CIMT Center for Medical Informatics and Telemedicine, School of Medicine, Universidad de Chile, Santiago, Chile; ^2^ University of Chile, Biomedical Neuroscience Institute (BNI), ICBM, Santiago, Chile; ^3^ Unidad de Investigación de Deterioro Cognitivo y Prevención de Demencia, Instituto Peruano de Neurociencias, Lima, Lima, Peru; ^4^ Memory and Neuropsychiatric Center (CMYN), Neurology Department, Hospital del Salvador and Faculty of Medicine, Universidad de Chile, Santiago, Chile; ^5^ Neuropsychology and Clinical Neuroscience Laboratory (LANNEC), Physiopathology Department ‐ ICBM, Neuroscience and East Neuroscience Departments, Faculty of Medicine, Universidad de Chile, Santiago, Chile; ^6^ University of Chile, Santiago, Chile; ^7^ CONICET, Buenos Aires, Argentina; ^8^ Cognitive Neuroscience Center (CNC), Universidad de San Andrés, Buenos Aires, Buenos Aires, Argentina; ^9^ Geroscience Center for Brain Health and Metabolism (GERO), Santiago, Metropolitana, Chile; ^10^ Neurology Unit, Hospital San José, Santiago, Chile; ^11^ Universidad de Chile, Santiago, Chile; ^12^ J.J. Aguirre's Clinical Hospital, University of Chile, Santiago, Chile; ^13^ University of Chile, Biomedical Neuroscience Institute (BNI), ICBM, Santiago, Metropolitana, Chile; ^14^ CIMT Center for Medical Informatics and Telemedicine, School of Medicine, Universidad de Chile, santiago, Chile; ^15^ Geroscience Center for Brain Health and Metabolism (GERO), Santiago, Chile; ^16^ Neurology Service, Department of Medicine, Clínica Alemana, Universidad del Desarrollo, Santiago, Región Metropolitana de Santiago, Chile; ^17^ Memory and Neuropsychiatric Center (CMYN) Neurology Department, Hospital del Salvador and Faculty of Medicine, University of Chile, Santiago, Region Metropolitana, Chile; ^18^ Neurology Department, Hospital del Salvador, University of Chile, Santiago, Región Metropolitana de Santiago, Chile

## Abstract

**Background:**

Functional impairment (FI) is a key component in diagnosing and monitoring the severity of neurodegenerative dementia (ND). It manifests in several dimensions, such as everyday cognition, mobility, social functioning, and activities of daily living (ADLs). However, traditional assessments have primarily focused on ADLs, often neglecting the other dimensions of functional phenotype.

This study aims to assess the utility of the Chilean multidimensional functional assessment (Ch‐MFA, see Figure 1), including ADLs, social behavior, mobility, and everyday cognition in differentiating Alzheimer's disease (AD) and frontotemporal dementia (FTD), and to predict their severity measured in the Clinical Dementia Rating (CDR).

**Method:**

308 patients were recruited in Peru, comprising 209 controls, 44 FTD, and 55 AD. Functional assessments include ADL (T‐ADLQ, DAD‐E), social behavior Blessed Dementia Rating Scale (BDR), mobility (WHODAS), everyday cognition (ECog), and CDR for severity. Data was normalized for each assessment, and groups were stratified regarding age and educational level, obtaining 72 controls, 24 FTD, and 45 AD for the predictive analysis. Random Forest (RF) models were trained using 70% of the data, while 30% was reserved for testing the prediction. Cross‐validation (20‐fold) and feature selection were used to reduce overfitting and identify a simpler set of questions.

**Result:**

The Ch‐MFA indicates a 0.81 ± 0.03 average f1‐score when differentiating the group (control, FTD, and AD), which is 0.12 ± 0.10 better than the best‐performing unidimensional assessment (T‐ADLQ) with only 14 questions (4 T‐ADLQ, 2 BDR, 4 ECog and 3 WHODAS). As for the severity, Ch‐MFA shows a 0.65 ± 0.17 average f1‐score when differentiating severities of 0.5, 1, and 2, increasing to 0.88 ± 0.06 when it's only between 1 and 2 with a total of 4 questions (2 T‐ADLQ, 2 ECog). See Figure 2.

**Conclusion:**

Our results show that the Ch‐MFA achieves better results than a unidimensional assessment. Interestingly, functional dimensions that distinguish types of dementia differed from those that predict their severity. Our results suggest that a multidimensional phenotype may better reflect the specificity of dementia subtypes.